# Prevalence of skin diseases in Taiwan prisons: a population-based study

**DOI:** 10.1186/s12889-023-15323-5

**Published:** 2023-03-10

**Authors:** Zhu Liduzi Jiesisibieke, Jiamin Lin, Yu-Chun Lin, Yi-Ying Hsiao, Tao-Hsin Tung

**Affiliations:** 1 Evidence-Based Medicine Center, Taizhou Hospital of Zhejiang Province Affiliated to Wenzhou Medical University, Linhai, 317000 Zhejiang China; 2 School of Public Health, The University of Hong Kong Li Ka Shing Faculty of Medicine, Hong Kong, SAR China; 3 Department of Plastic Surgery, Taizhou Hospital of Zhejiang Province Affiliated to Wenzhou Medical University, Taizhou, 318000 Zhejiang PR China; 4 Department of Public Health, College of Health Science, Kaohsiung Medical University, Kaohsiung, Taiwan

**Keywords:** Taiwan, Prison, Skin disease, Sex difference, Age difference

## Abstract

**Background:**

The prevalence of skin diseases among prisoners in Taiwan has rarely been investigated. This study aimed to estimate the prevalence of skin diseases by sex in a sample of prisoners in Taiwan.

**Methods:**

We included 83,048 participants from the National Health Insurance Program. The outcomes were measured using the clinical version of the International Classification of Diseases, Ninth Revision. For prevalence, we presented absolute values as well as percentages. We also conducted an *X*^*2*^ test to assess sex differences and age group differences in the percentages of skin and subcutaneous tissue diseases.

**Results:**

The prevalence of skin diseases was 42.25%, higher than that in the general population. The prevalence of skin diseases among male prisoners was higher than that among female prisoners (*p* < 0.001), and the prevalence of skin diseases among prisoners who were ≤ 40 was higher than that among prisoners who were > 40. Among all cases diagnosed with skin disease, the top three diseases were contact dermatitis and other types of eczema, cellulitis and abscess, pruritus, and related conditions. Male prisoners had a significantly higher prevalence of all types of skin diseases than female prisoners.

**Conclusions:**

Skin diseases are common in prisoners in Taiwan. Therefore, early prevention and appropriate treatment are needed. Male-specific skin products are also needed, given the differences in the prevalence of skin diseases among male and female prisoners.

## Key Points

1. The prevalence of skin diseases is higher among prisoners than in the general population in Taiwan.
2. Contact dermatitis and other eczemas were the most prevalent skin diseases among both male and female prisoners.
3. The results suggest the need for early prevention and appropriate treatment of skin diseases in the criminal justice system in Taiwan

## Background

Skin disease is a common disorder that causes non-fatal disability worldwide and was estimated to account for 1.79% of the total health burden of disease in 2013 [1]. In Asia, inflammatory dermatoses are a significant health issue, particularly in some underdeveloped countries, and infectious diseases cannot be neglected based on clinical research published between 1990 and 2017 [2]. Previous studies have provided evidence of skin disease profiles among African inmates [3], prisoners in India [4], Nigeria [5], female prisoners in Turkey [6], and male prisoners in Lazio [7]. However, it is difficult to compare the morbidity of skin-related diseases in different areas because the measurement methods are not consistent: some of the measurements are based on disease history and clinical findings [3], pathological examination [5], examination under daylight [4], or with the indication' dermatology examination' without sufficient details [6, 7]. However, there is a shortage of data on the skin health of female inmates.

In Taiwan, the prevalence of skin disease was estimated to be 35.5%. The general population of Taiwan has easy access to dermatological health services due to a single-payer health system; 52.6% of patients directly visit dermatologists for treatment [8]. Skin diseases profoundly affect patients' social activities, personal connections, and psychological status [9]. Therefore, it is essential to identify skin diseases and provide dermatological treatment. Additionally, from a clinical viewpoint, diagnostic methods have changed in dermatology; for example, dermatoscopy has modified the incidence and prevalence of pigmented lesions [10, 11] by making it easier to screen and surveil for skin diseases.

In prison, due to safety considerations, poor environment, and hygiene, it is not convenient for prisoners to receive timely treatment. The burden of mental, infectious, and chronic diseases and cognitive disability is higher among prisoners [12]. It is important to understand the specific skin disease profile which is particularly relevant to the prison environment, and that can be improved. Given the need for equal healthcare for prisoners and inadequate attention to them, it is of great public interest to focus on prisoners' health issues. Therefore, using a large sample size, we conducted this cross-sectional descriptive study to present the skin disease profile of prisoners in Taiwan to facilitate future related studies.

## Methods

### Data source

Taiwan's National Health Insurance (NHI) system covers 99% of the population. The National Health Insurance Research Database (NHIRD) was developed based on information collected from the population in Taiwan [13]. The International Classification of Diseases 9th revision Clinical Modification (ICD-9-CM) was used in the NHIRD from 1995 to 2016. The ICD-10 has been used since 2017 [14]. Details of this data source and design are described elsewhere [15]. Briefly, information about prisoners from January 1st, 2013, to December 31st, 2013, was presented in a subset of the NHIRD. Our study included information from 2013 (January 1st to December 31st); therefore, we used the ICD-9 to present the results. This study was approved by the Institutional Review Board of Cheng-Hsin General Hospital (CHGH-IRB: (471) 104–07). All the procedures were in accordance with the required guidelines, and all the participants provided informed consent.

### Study population

This study included 83,048 prisoners. Most were male (89.59%), and 10.41% were women. We measured the outcomes based on the ICD-9 codes (680–709). Among them, ICD9_680-686 represents infections of the skin and subcutaneous tissue; ICD9_690-698 represents other inflammatory conditions of the skin and subcutaneous tissue; ICD9_700-709 represents other diseases of the skin and subcutaneous tissue. To maintain strict algorithms, only patients diagnosed at least three times in one group were treated as disease cases [15].

### Statistical analysis

SAS (version 9.4; SAS Institute Inc., Cary, NC, USA) for Windows was used to perform all analyses in this study. We presented the mean and standard deviation (SD) of age. For prevalence, we presented absolute values as well as percentages. We also conducted an *X*^*2*^ test to assess sex differences and age group differences in the percentages of skin and subcutaneous tissue diseases.

## Results

The demographic information of the participants is shown in Table 1. The mean ages of female and male prisoners were 38.65 (SD: 11.79) and 41.50 (SD: 11.43), respectively. The mean ages for females and males with skin and subcutaneous tissue diseases were 38.12 (SD: 11.51) and 41.38 (SD: 11.97), respectively. Prisoners with skin and subcutaneous tissue disease had 1.3 times higher medicine service time per year.

**Table 1:** Demographics of the participating sample by sex (Taiwan, 2013)

	Total		Diseases of the skin and subcutaneous tissue	
	Female (*n* = 8,643)	Male (*n* = 74,405)	Female (*n* = 3,215)	Male (*n* = 31,876)
Age				
Mean (standard deviation)	38.65(11.79)	41.50(11.43)	38.12(11.51)	41.38 (11.97)
Range (min–max)	2–88	2–103	2–88	2–99
Medicine Service Times (a year)				
Mean (standard deviation)	18.17(15.83)	13.27(13.16)	23.03(17.68)	17.72(14.44)
Range (min–max)	1–228	1–369	2–228	2–369

The prevalence of skin and subcutaneous tissue diseases according to sex is shown in Table 2. The prevalence of skin and subcutaneous tissue disease among females was 3.87%, while the prevalence in males was much higher (38.38%, *p* < 0.001). Among female prisoners, the top three skin and subcutaneous tissue diseases were contact dermatitis and other types of eczema (2.27%), diseases of the sebaceous glands (0.93%), and urticaria (0.68%). The top three skin and subcutaneous tissue diseases in male prisoners were contact dermatitis and other types of eczema (25.42%), other cellulitis and abscess (7.8%), and pruritus and related conditions (7.36%).

**Table 2:** Prevalence of skin and subcutaneous tissue diseases by sex using a survey of 2013 claims data from the Taiwan National Health Insurance program (*N* = 83,048, Taiwan, 2013)

	Female			Male		
	n	%	mean age (S.D.)	n	%	mean age (S.D.)
**Total prisoners**	8,643	10.41	38.65(11.79)	74,405	89.59	41.50(11.43)
ICD9_680-709 Diseases of the skin and subcutaneous tissue	3215	3.87	38.12(11.51)	31,876	38.38	41.38(11.97)
**ICD9_680-686 Infections of skin and subcutaneous tissue**						
ICD9_680-Carbuncle and furuncle	117	0.14	37.44(9.61)	4507	5.43	39.36(11.76)
ICD9_681-Cellulitis and abscess of finger and toe	105	0.13	41.67(12.79)	1161	1.40	40.10(11.87)
ICD9_682- Other cellulitis and abscess	303	0.36	40.46(11.43)	6481	7.80	40.87(11.72)
ICD9_683-Acute lymphadenitis	16	0.02	38.75(12.40)	65	0.08	41.78(9.77)
ICD9_684-Impetigo	6	0.01	40.17(13.44)	337	0.41	37.87(11.64)
ICD9_685-Pilonidal cyst	1	< 0.01	-	22	0.03	41.86(8.72)
ICD9_686-Other local infections of skin and subcutaneous tissue	168	0.20	38.42(11.52)	3038	3.66	41.03(11.41)
**ICD9_690-698 Other inflammatory conditions of skin and subcutaneous tissue**						
ICD9_690-Erythematosquamous dermatosis	170	0.20	37.62(9.01)	1674	2.02	41.99(11.73)
ICD9_691-Atopic dermatitis and related conditions	82	0.10	33.66(16.01)	1145	1.38	42.55(13.39)
ICD9_692-Contact dermatitis and other eczema	1883	2.27	38.46(11.59)	21,107	25.42	41.44(12.16)
ICD9_693-Dermatitis due to substances taken internally	57	0.07	37.79(9.46)	386	0.46	43.91(12.88)
ICD9_694-Bullous dermatoses	0	-	-	62	0.07	42.30(13.04)
ICD9_695-Erythematous conditions	18	0.02	38.89(9.05)	78	0.09	46.01(12.35)
ICD9_696-Psoriasis and similar disorders	32	0.04	40.84(9.61)	485	0.58	44.93(10.95)
ICD9_697-Lichen	3	< 0.01	56(5.57)	13	0.02	44.92(11.56)
ICD9_698-Pruritus and related conditions	435	0.52	39.43(12.90)	6116	7.36	43.30(11.94)
**ICD9_700-709 Other diseases of skin and subcutaneous tissue**						
ICD9_700-Corns and callosities	12	0.01	37.67(6.01)	84	0.10	37.46(10.53)
ICD9_701-Other hypertrophic and atrophic conditions of skin	29	0.03	38.66(10.85)	140	0.17	40.71(13.31)
ICD9_702-Other dermatoses	38	0.05	43.71(11.17)	44	0.05	46.80(12.60)
ICD9_703-Diseases of nail	7	0.01	32.71(11.76)	33	0.04	42.12(13.33)
ICD9_704-Diseases of hair and hair follicles	325	0.39	34.59(9.27)	3602	4.34	38.36(10.98)
ICD9_705-Disorders of sweat glands	197	0.24	36.90(9.46)	795	0.96	38.27(10.98)
ICD9_706-Diseases of sebaceous glands	776	0.93	33.85(8.27)	2071	2.49	33.60(10.72)
ICD9_707-Chronic ulcer of skin	3	< 0.01	46.33(10.69)	230	0.28	48.28(13,27)
ICD9_708-Urticaria	565	0.68	39.16(10.98)	4294	5.17	41.58(11.81)
ICD9_709-Other disorders of skin and subcutaneous tissue	70	0.08	37.57(11.72)	301	0.36	43.88(12.75)

The prevalence of the most frequent skin and subcutaneous tissue diseases by sex is shown in Table 3. The diseases with the highest prevalence were contact dermatitis and other types of eczema (27.68%), other cellulitis and abscess (8.17%), and pruritus and related conditions (7.89%). Almost half (42.25%) of prisoners had skin and subcutaneous tissue diseases. There was a significant difference between females and males in the prevalence of all categories of skin and subcutaneous tissue diseases.

**Table 3:** Prevalence of most common skin and subcutaneous tissue diseases among prisoners by sex (*N* = 83,048, Taiwan, 2013)

	Total	Female		Male		*P* for *X2* test
	%	n	%	n	%	
Total prisoners		8643	10.41	74,405	89.59	
ICD9_680-709 Diseases of the skin and subcutaneous tissue	42.25	3215	3.87	31,876	38.38	< .001
ICD9_692-Contact dermatitis and other eczema	27.68	1883	2.27	21,107	25.42	< .001
ICD9_682- Other cellulitis and abscess	8.17	303	0.36	6481	7.80	< .001
ICD9_698-Pruritus and related conditions	7.89	435	0.52	6116	7.36	< .001
ICD9_708-Urticaria	5.85	565	0.68	4294	5.17	0.004
ICD9_680-Carbuncle and furuncle	5.57	117	0.14	4507	5.43	< .001
ICD9_704-Diseases of hair and hair follicles	4.73	325	0.39	3602	4.34	< .001

The prevalence of skin and subcutaneous tissue diseases according to age is shown in Table 4. The prevalence of skin and subcutaneous tissue disease among prisoners who were ≤ 40 was 22.17%, while the prevalence in males was much higher (20.08%, *p* < 0.001). Among prisoners who were ≤ 40, the top three skin and subcutaneous tissue diseases were contact dermatitis and other types of eczema (14.41%), other cellulitis and abscesss (4.33%), and pruritus and related conditions (3.62%). The top three skin and subcutaneous tissue diseases in prisoners who were > 40 were contact dermatitis and other types of eczema (13.27%), pruritus and related conditions (4.26%), and other cellulitis and abscess (3.84%).

**Table 4:** Prevalence of diseases of the skin and subcutaneous tissue by age group using a survey of 2013 claims data from the Taiwan National Health Insurance program (*N* = 83,048, Taiwan, 2013)

	≤ 40		> 40	
	n	%	n	%
**Total prisoners**	42,684	51.40	40,364	48.60
ICD9_680-709 Diseases of the skin and subcutaneous tissue	18,413	22.17	16,678	20.08
**ICD9_680-686 Infections of skin and subcutaneous tissue**				
ICD9_680-Carbuncle and furuncle	2725	3.28	1899	2.29
ICD9_681-Cellulitis and abscess of finger and toe	714	0.86	552	0.66
ICD9_682- Other cellulitis and abscess	3597	4.33	3187	3.84
ICD9_683-Acute lymphadenitis	39	0.05	42	0.05
ICD9_684-Impetigo	228	0.27	115	0.14
ICD9_685-Pilonidal cyst	10	0.01	13	0.02
ICD9_686-Other local infections of skin and subcutaneous tissue	1747	2.10	1459	1.76
**ICD9_690-698 Other inflammatory conditions of skin and subcutaneous tissue**				
ICD9_690-Erythematosquamous dermatosis	968	1.17	876	1.05
ICD9_691-Atopic dermatitis and related conditions	621	0.75	606	0.73
ICD9_692-Contact dermatitis and other eczema	11,969	14.41	11,021	13.27
ICD9_693-Dermatitis due to substances taken internally	212	0.26	231	0.28
ICD9_694-Bullous dermatoses	32	0.04	30	0.04
ICD9_695-Erythematous conditions	38	0.05	58	0.07
ICD9_696-Psoriasis and similar disorders	192	0.23	325	0.39
ICD9_697-Lichen	2	< 0.01	14	0.02
ICD9_698-Pruritus and related conditions	3101	3.62	3541	4.26
**ICD9_700-709 Other diseases of skin and subcutaneous tissue**				
ICD9_700-Corns and callosities	60	0.07	36	0.04
ICD9_701-Other hypertrophic and atrophic conditions of skin	98	0.12	71	0.09
ICD9_702-Other dermatoses	38	0.05	44	0.05
ICD9_703-Diseases of nail	22	0.03	18	0.02
ICD9_704-Diseases of hair and hair follicles	2489	3.00	1438	1.73
ICD9_705-Disorders of sweat glands	621	0.75	371	0.45
ICD9_706-Diseases of sebaceous glands	2269	2.73	578	0.70
ICD9_707-Chronic ulcer of skin	66	0.08	167	0.20
ICD9_708-Urticaria	2530	3.05	2329	2,80
ICD9_709-Other disorders of skin and subcutaneous tissue	176	0.21	195	0.23

The prevalence of the most frequent skin and subcutaneous tissue diseases by age group is shown in Table 5. There was a significant difference between females and males in the prevalence of almost all categories of skin and subcutaneous tissue diseases, except urticaria.

**Table 5:** Prevalence of most common skin and subcutaneous tissue diseases among prisoners by age group (*N* = 83,048, Taiwan, 2013)

	Total	≤ 40		> 40		*P* for *X2* test
	%	n	%	n	%	
**Total prisoners**		42,684	51.40%	40,364	48.60%	
ICD9_680-709 Diseases of the skin and subcutaneous tissue	42.25	18,413	22.17	16,678	20.08	< .001
ICD9_692-Contact dermatitis and other eczema	27.68	11,969	14.41	11,021	13.27	0.018
ICD9_682- Other cellulitis and abscess	8.17	3597	4.33	3187	3.84	0.005
ICD9_698-Pruritus and related conditions	7.89	3010	3.62	3541	4.26	< .001
ICD9_708-Urticaria	5.85	2530	3.05	2329	2.80	0.334
ICD9_680-Carbuncle and furuncle	5.57	2725	3.28	1899	2.29	< .001
ICD9_704-Diseases of hair and hair follicles	4.73	2489	3.00	1438	1.73	< .001

## Discussion

Although prisoners may be easily punished, they have equal rights to health services. The World Health Organization has suggested reducing any "avoidable or unfair" health differences, stating that prisoners are entitled to have equal access to health services [16]. Therefore, this study was conducted to understand the skin and subcutaneous tissue disease profiles among prisoners. Previously, we conducted a study to understand the mental health of prisoners [15], which can be a part of a series study. To our knowledge, this is the first study to describe skin diseases among prisoners in Taiwan. The main finding is that prisoners have a higher prevalence of skin diseases (42.25%) than the general population (35.5%) [8]. The results also suggest that male prisoners had a higher prevalence of skin and subcutaneous tissue diseases than female prisoners.

The prevalence of skin and subcutaneous tissue diseases in our study was estimated to be 42.25%, indicating that staff working in prison should pay attention to the exact number of prisoners with skin and subcutaneous tissue diseases. A study conducted in France found that more than half of patients with skin diseases argued that detention was directly associated with skin diseases [17]. Possible reasons include overcrowding, poor hygiene, etc. [3, 5, 18]. For example, although the quality of some of the included studies was poor, one systematic review supported the association between prison population density and infectious and communicable diseases [19]. Overcrowding and poor prison environments are also related to the mental status of prisoners [20]. The health issues of prisoners are not just a problem of the prison itself; it is also a problem of society since more than 95% of prisoners will eventually return to normal life [21], and their health issues may become a community or even a societal burden.

Male prisoners had a higher prevalence of skin and subcutaneous tissue diseases than female prisoners. In the general population, due to skin structure and function, the immune system, and sex hormones in males and females, sex differences exist in the prevalence of skin and subcutaneous tissue diseases [22]. Sex differences make it important to produce and prepare male-specific skin products for males [23]. Based on our findings in Taiwan prisons, the number of males outweighed that of females, and dermatologists should consider more products for male prisoners.

A multicenter study in Italy found that about 40% of patients with skin diseases changed or even stopped therapy without consulting physicians due to poor knowledge of COVID-19 [24]. The results implied that the COVID-19 pandemic had negatively affected skin health-related treatments. Additionally, driven by the challenge of COVID-19 prevention, patients with chronic diseases may be missed or ignored, especially in some low- and middle-income countries/regions [25]. In prisons, healthcare professionals should maintain health services for prisoners with chronic diseases such as skin diseases.

### Methodological considerations

One obvious advantage of this study is the large sample size, which enabled us to obtain a general picture of skin and subcutaneous tissue diseases among prisoners in Taiwan. In addition, we included information from the Taiwan region instead of information from only one prison. The outcome was measured based on unified ICD codes; therefore, measurement bias was largely avoided. Furthermore, the comparability of our results was improved. We acknowledge that this study had some limitations. First, this was a descriptive cross-sectional study; therefore, we could not identify any risk factors or conclude any causality based on the analysis. Future longitudinal studies with longer follow-up periods are needed to identify potential exposure to skin and subcutaneous tissue diseases. Second, all the participants were from Taiwan prisons; therefore, the generalizability to other places or races may be limited. Finally, with a short follow-up of 1-year, this study could not provide a trend of skin diseases among prisoners in Taiwan.

## Conclusions

In this study, we observed a higher prevalence of skin and subcutaneous tissue diseases among prisoners in Taiwan than in the general population. We also found a sex difference in the prevalence of skin and subcutaneous tissue diseases among prisoners in Taiwan. These findings emphasize the importance of identifying and treating skin diseases in Taiwan prisons. Further, it provides a reminder to enhance health equality in prisons in Taiwan.

## Data Availability

All data generated or analyzed during this study are included in this published article.
